# Differential regulation of immune responses and macrophage/neuron interactions in the dorsal root ganglion in young and adult rats following nerve injury

**DOI:** 10.1186/1744-8069-5-70

**Published:** 2009-12-10

**Authors:** David Vega-Avelaira, Sandrine M Géranton, Maria Fitzgerald

**Affiliations:** 1 Department of Neuroscience, Physiology and Pharmacology, University College of London, Gower Street, London, WC1E 6BT, UK; 2 Department of Cell and developmental Biology, University College of London, Gower Street, London, WC1E 6BT, UK

## Abstract

**Background:**

Neuropathic pain is an apparently spontaneous experience triggered by abnormal physiology of the peripheral or central nervous system, which evolves with time. Neuropathic pain arising from peripheral nerve injury is characterized by a combination of spontaneous pain, hyperalgesia and allodynia. There is no evidence of this type of pain in human infants or rat pups; brachial plexus avulsion, which causes intense neuropathic pain in adults, is not painful when the injury is sustained at birth. Since infants are capable of nociception from before birth and display both acute and chronic inflammatory pain behaviour from an early neonatal age, it appears that the mechanisms underlying neuropathic pain are differentially regulated over a prolonged postnatal period.

**Results:**

We have performed a microarray analysis of the rat L4/L5 dorsal root ganglia (DRG), 7 days post *spared nerve injury*, a model of neuropathic pain. Genes that are regulated in adult rats displaying neuropathic behaviour were compared to those regulated in young rats (10 days old) that did not show the same neuropathic behaviour. The results show a set of genes, differentially regulated in the adult DRG, that are principally involved in immune system modulation. A functional consequence of this different immune response to injury is that resident macrophages cluster around the large A sensory neuron bodies in the adult DRG seven days post injury, whereas the macrophages in young DRG remain scattered evenly throughout the ganglion, as in controls.

**Conclusions:**

The results show, for the first time, a major difference in the neuroimmune response to nerve injury in the dorsal root ganglion of young and adult rats. Differential analysis reveals a new set of immune related genes in the ganglia, that are differentially regulated in adult neuropathic pain, and that are consistent with the selective activation of macrophages around adult, but not young large A sensory neurons post injury. These differences may contribute to the reduced incidence of neuropathic pain in infants.

## Background

Physiological pain is an acute experience that results from the activation of peripheral nociceptors in injured and inflamed tissues and which normally passes when the stimulus ceases. In contrast, neuropathic pain is an apparently spontaneous experience triggered by abnormal physiology of the peripheral or central nervous system, which evolves with time. Neuropathic pain arising from peripheral nerve injury is characterized by a combination of spontaneous pain, hyperalgesia and allodynia and as it is poorly relieved by conventional analgesics, it is a significant clinical problem [1]. Neuropathic pain is less common in children than in adults. Although it has been reported in very young paediatric patients [2], there is no evidence of this type of pain in infancy; brachial plexus avulsion, which causes intense neuropathic pain in adults, is not painful when the injury is sustained at birth [3]. Consistent with this, persistent mechanical allodynia does not develop in spared nerve injury (SNI) and chronic constriction injury (CCI) rat models of neuropathic pain, if the injury is performed younger than 4 weeks of age [4]. Furthermore, in the spinal nerve ligation model (SNL), neuropathic symptoms resolve faster in younger animals [5]. Since infants are capable of nociception from before birth and display both acute and chronic inflammatory pain behaviour from an early neonatal age [6], it appears that the mechanisms underlying neuropathic pain are differentially regulated over a prolonged postnatal period.

The mechanisms underlying neuropathic pain in adults involve molecular and cellular changes in both the peripheral and central nervous system to produce the characteristic intensity and prolonged time course of the pain [7,8]. Increased focus has been placed upon the role of neuroimmune interactions in animal models of persistent pain suggesting that the major stimulus for the underlying neuronal hypersensitivity in chronic pain states involves activation of glial cells, which when stimulated, increase production of a host of inflammatory/algesic mediators, such as cytokines and chemokines [9,10]. In the peripheral nervous system, primary afferent neurons in the dorsal root ganglion (DRGs) [11] interact with two main types of non-neuronal cells in chronic pain states: satellite cells which surround neurons and control their chemical environment [12] and resident macrophages that are activated by nerve damage and inflammation [13-15].

Our overall aim is to discover why nerve injury induced neuropathic pain does not occur or is rapidly resolved in young animals, while it is intense and prolonged in adults. In the present study, we hypothesise that the postnatal maturation of the neuropathic pain response in young animals arises, at least in part, from developmental changes in the response of the dorsal root ganglion to nerve injury. To test our hypothesis, we analysed and compared transcriptional changes in young and adult DRGs after neuropathic nerve injury or sham controls, using high throughput screening with the Affymetrix^® ^array rat203_v2. Our results reveal a new set of genes that implicate the peripheral immune system in the regulation of neuropathic pain. Immunohistochemical analysis of the dorsal root ganglia after nerve injury confirmed a selective pattern of macrophage activation that is specific for injured neurons in adult, but not young rats.

## Materials and methods

### Surgery

Experiments were performed on adult (2 months old) male Sprague-Dawley rats and rat pups (10 days postnatal age, P10) and carried out in accordance with the United Kingdom Animal (Scientific Procedures) Act 1986. Spared nerve injury and sham surgery were performed under halothane general anaesthesia with antiseptic conditions as described previously [16]. After skin preparation, the sciatic nerve and its three terminal branches were exposed in the upper lateral thigh. Axotomy and ligation of the common peroneal and tibial branches was performed under direct vision, leaving the sural nerve intact. Retrograde tracer (4 μL of 4% Fluorogold, Fluorochrome^®^, LLC) was injected into the cut end of the severed nerves to identify injured neurons [17]. In adult rats after SNI surgery, 92.4% ± 1 (SE) of Fluorogold labelled neurons were also labelled with ATF-3 (which is known to mark damaged and regenerating neurons [18]) (Additional file 1). Muscle and skin were closed in two layers. In the sham operation, the procedures were the same but the nerves were only exposed and not cut or ligated. In all cases great care was taken not to stretch the nerve, or its branches, nor to damage the intact nerves.

After surgery, animals were returned to their cages and litters and maintained on a 12 hour light/dark cycle at constant ambient temperature with free access to food and water for 7 days post surgery until tissue collection.

### RNA extraction

Seven days post surgery, animals were sacrificed with 100 mg/kg of Euthanal^®^, fresh DRGs were collected, snap frozen on liquid nitrogen and stored at -80°C until RNA extraction. Both L4 and L5 DRGs ipsilateral to the lesion were collected and pooled from 3 animals per adult experimental group and 4 animals per P10 group, (i.e. n = 6 DRGs per adult sample, n = 8 per P10 sample) to minimise inter-animal differences. RNA was extracted using Trizol^® ^reagent (Invitrogen^®^) and a QIAshredder column (Qiagen^®^) for homogenisation according to manufacturer's protocols. The RNA was cleaned by an "RNA purification column" (Qiagen^®^) and a final volume of 50 μL obtained. The amount of RNA was measured with a *NanoDrop ND-1000 Spectrophotometer *(NanoDrop Technologies, Inc.) and its quality assessed with *Experion RNA HighSens StdSens analysis kit *(Bio-Rad^®^). Then, the RNA was precipitated and dissolved to obtain a final concentration of 1 μg/μL and stored at -80°C for cRNA synthesis or amplification by quantitative real-time polymerase chain reaction (qPCR).

### cRNA synthesis

#### First strand cDNA synthesis

2 g (for qPCR) or 10 μg (microarray analysis) of total RNA and 100 pmol/μL of the T7(dT)24 primer (5'-GGCCAGTGAATTGTAATACGACTCACTATAGGGA GGCGGTTTTTTTTTTTTTTTTTTTTTTTT-3') were combined for annealing at 60°C for 10 mins and chilled on ice for 2 mins. Then, the SuperScript III kit (Invitrogen^®^) was added according to manufacturer instructions to synthesize the cDNA.

#### Second strand cDNA synthesis

the whole amount of the cDNA produced was used as template for the second strand synthesis plus 10 units of *E. coli *DNA ligase, 40 units of *E. coli *DNA polymerase (Invitrogen^®^), 3 μL of 10 mM dNTPs and 2 units of Rnase H (Invitrogen^®^). The reaction was stopped by addition of 10 μL of 0.5 M EDTA. The double stranded cDNA was then purified and precipitated and then resuspended in 10 μL of nuclease free water.

#### Synthesis of biotin-labelled cRNA

we used the *Enzo-Bioarray *kit (Enzo Life Sciences, Inc) for synthesis of the cRNA according to the manufacturer protocol. The resulting cRNA was purified with Rneasy kit (Qiagen^®^). The concentration was measured using a spectrophotometer and 20 μg of each sample were fragmented in 200 mM tris-acetate (pH= 8.1), 500 mM potassium acetate and 150 mM magnesium acetate for 35 mins at 95°C, then chilled on ice and stored at -80°C until microarray hybridisation.

### Microarray analysis

#### cRNA hybridisation

For mRNA high-throughput screening we used *GeneChip *^® ^*Rat expression 230 version 2.0 *arrays and *GeneChip*^®^*Hybridization, Wash and stain *kit from Affymetrix^®^. The hybridisation protocol was in *49/64 *formats, appropriate for the selected array. The control oligonucleotides were: *B2 *and the *Eukaryotic bioB, bioC, bioD and cre*. Then, the samples were washed and stained on the *Fluidics Station 450 *(Affymetrix^®^) monitored with the *GeneChip*^®^*Operating Software *(Affymetrix^®^) (protocol FS450_003 appropriate for the rat 230 v2.0 array) and scanned with *GeneChip Scanner 3000 *(Affymetrix^®^).

#### Array statistical analysis

the ".CEL" files generated with *GeneChip Scanner 3000 *(Affymetrix^®^) (see additional file 2 for details) were acquired by "R environment for statistical computing and graphics language software" (the GNU project) using the BioConductor^® ^package [19]. The expression data was normalised by GC-Robust Multi-array Average (GC-RMA) background adjustment. *Limma *analysis [20] was used as a lineal method for contrasting the groups and identifying specific genes for each experimental group.

**Figure 1:**
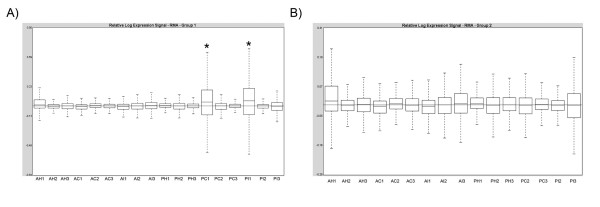
**Quality assessment of the microarrays**. The figure shows the relative expression signal of the RMA-normalised microarrays before (a) and after (b) removal of the outliers (*). A: adult; P: 10 day old rat; I: spared nerve injury group H: sham group and C: contralateral group (all 7 days after surgery).

The quality of the arrays was assessed with *Affymetrix Expression Console™ Software Version 1.0 *(Affymetrix^®^) using an n = 3 per experimental group. Two samples out of 18 were outliers (Figure 1), one from P10 contralateral group and one from the P10 injured group. These two samples were removed from further analysis.

### Quantitative Real-Time PCR

Specific primers for the gene candidates obtained from the microarray study were designed and synthesized by Sigma-Genosys (Table 1). An SYBR green-based system (Applied Biosystems) was used for amplification and detection. 1 μL of cDNA product per sample, 12.5 μl of Power SYBR green PCR Master Mix and 200 nM/primer was used for cocktail reaction. The amplification protocol was: step 1 at 95°C for 5', step 2 at 95°C for 45", step 3 at 60°C 1' step 4 at 70°C 1', step 5 plate reading, 39 cycles from step 2 to 4 and a final melting curve from 60°C to 90°C with reading every 0.5°C and 1" hold. The reaction plate was then placed in a *DNA Engine Pelter *thermal cycler with a *Chromo 4 Real-Time PCR *detector (Bio-Rad^®^). The amplification process was monitored with Opticon Monitor v3.1.32 software (MJ Genework Inc).

**Table 1:** Primers sequences for quantitative Real-Time PCR.

Serine (or cysteine) inhibitor, clade A, member 3N	Sens	CAGGCAATGCCCTGTTTATT
	Antisens	CACGAGACTGCTGGAAATCA
**Serine (or cysteine) inhibitor, clade B, member 2**	Sens	CACCACGCTTGGGAGATTAT
	Antisens	TTCATTGGCACATTCAAGGA

**Interleukin-6**	Sens	CTTCCAGCCAGTTGCCTTCTTG
	Antisens	TGGTCTGTTGTGGGTGGTATCC

**Mitogen-activated protein kinase kinase 6**	Sens	GTTGACTTTACCTCACAGTGCTTGAAG
	Antisens	TCCCCGAGTATCAGTTTTACGAAAGAC

**Chemokine (C-C motif) ligand 2**	Sens	ATGCAGTTAATGCCCCACTC
	Antisens	TTCCTTATTGGGGTCAGCAC

Inositol 1,4,5-trisphosphate 3-kinase C	Sens	TGATGGCTCCCTCATAGGAC
	Antisens	TCACCAAGTCTCTGGGCTCT

**Matrix metallopeptidase 16**	Sens	GGAATTGGAGGCGATACTCA
	Antisens	CCAGCTCATGGACTGCTACA

**Monoamino oxidase A**	Sens	AGCAAGACACGCTCAGGAAT
	Antisens	CCACAGGGCAGATACCTCAT

**Glyceraldehyde-3-phosphate dehydrogenase**	Sens	ACTCTACCCACGGCAAGTTC
	Antisens	GGTGGTGAAGACGCCAGTAG

**Caspase 3**	Sens	CCCTCTCATTTAAATCAAATCCG
	Antisens	GTTCCTAATTAAATTTCATTTCAACTACC

**Toll like receptor 4**	Sens	TCTCTTTAGACCTGTCTTTAAACC
	Antisens	AAGGCACATTTTCAGTACATTTG

**Matrix metallopeptidase 3**	Sens	TTGATGAGAAGAAACAATCCATG
	Antisens	CGCTGAAGAAGTAAAGAAACC

**Complement component3**	Sens	GAGCAAGCTGTGCCACAATG
	Antisens	GGTCTTGTACACGTAGTCCACTC

**cAMP responsive element modulator**	Sens	ATGAAACTGATGAGGAGACTGACC
	Antisens	GCTCGGATCTGGTAAGTTGGC

**Colony stimulating factor 1 (macrophage)**	Sens	ATCCCGTTTGCTACCTAAAG
	Antisens	AGCTGTTCAGTTTCATAGAGAG

**Chloride intracellular channel 1**	Sens	TCCACGTTGCTACATAATGG
	Antisens	CCCTTGTCCTCTAATCTTTCC

**Purinergic receptor P2Y, G-protein coupled, 14**	Sens	ACATACAGTGCATGGAACTC
	Antisens	CAGAAAATGCTCACGAAGAC

The DNA products from the qPCRs were sequenced on an AB PRISM 3100-Avant automated DNA capillary sequencer (AB Applied Biosystems, Lingley House, 120, Birchwood Boulevaid, Warrington) using BigDye Terminator v1.1 Cycle Sequencing chemistry (AB Applied Biosystems). Sequencing reactions were performed and precipitated in accordance with the manufacturer's instructions using the same *sense *or *forward *primer that was used for the qPCR amplification (Table 1). The reactions were resolved on a 50 cm standard sequencing capillary, running optimal 3100 POP-6TM polymer (AB Applied Biosystems). Sequence data was extracted and analysed using AB PRISM 3100-Avant Data Collection Software v2.0 and DNA Sequencing Analysis Software v5.1.1 respectively (AB Applied Biosystems).

### Immunostaining

Seven days post surgery rats were overdosed with 100 mg/kg of Euthanal^® ^and perfused through the heart with 200 ml heparin-saline, followed by 200 mL 4% paraformaldehyde in 0.1 M phosphate buffer (PB). The lumbar ganglia L4 and L5 were removed and postfixed in 4% paraformaldehyde for 1 h at 4°C followed by immersion in 30% sucrose in 0.1 M of PB at 4°C for at least two days before sectioning. Serial, 20 μm cryostat sections were cut and stained after blocking in 10% normal goat serum, 0.3% Triton X in 1× PBS using primary antibodies: Rabbit anti-IBA-1 (1/1000, Dako^®^), mouse anti NF200 (1/1000, Chemicon^®^), rabbit anti ATF-3 (1/500, Santa Cruz^®^), mouse anti CD68/ED-1 (1/200. Serotec^®^), mouse anti NG2 (1/500, Millipore^®^) and rabbit-anti-CGRP (1/500 Chemicon^®^). The antibodies were incubated overnight at room temperature in 3% normal goat serum, 0.3% Triton X in 1× PBS. Secondary antibodies were goat anti-mouse (Alexa-593^®^) and goat anti-rabbit (Alexa-488^®^) at 1/200 dilution in 3% normal goat serum, 0.3% Triton X in 1× PBS. Antibody excess was removed by 3 washes in PBS for 10' at RT. The isolectin IB-4 (*Bandeira simplicifolia*) labelled with FITC (Sigma^®^) was used to identify the non-peptidergic neurons. After staining, the sections were kept for 16 h in the dark to normalize the background and then microscope images acquired at 10× magnification with OpenLab^® ^software at a constant exposure time of 1.02".

### Data Analysis for immunohistochemistry and qPCR

The images generated by immunohistochemistry were analysed with *Image J 1.36 *(NHS) software, n = 3 rats per experimental group, n = 4-6 sections per animal and n = 200 cells counted per section. The sections were from the centre of the ganglion and 160 μm apart to ensure that the same cell was not counted twice (large neurons may have a diameter up to 100 μm). Cells were counted as having macrophage 'ring-like' structures when the perimeter of the neuron was surrounded by macrophages which were closely adhered to the neuronal surface, as observed in merged images from IBA-1 and NF200 immunostaining. Data was pooled from animals in the same experimental group.

Pooled qPCR data from each experimental group (n = 3 samples per group) were plotted as "mean intensity normalized" (MIN) ± standard deviation (SD). The normalisation was calculated as the ratio with the housekeeping gene, Gapdh. All data was exported to SigmaStat, 2.03^® ^(SPSS Inc^®^) for statistical analysis and plotted with SigmaPlot^® ^(SPSS Inc^®^).

## Results

### Differential microarray screening of DRG following nerve injury reveals 'neuropathic pain' specific genes

We performed high throughput screening of the mRNA regulation in L4/L5 dorsal root ganglia following spared nerve injury (SNI) or sham injury in adult and young rats using the *Affymetrix-rat genome 230 2.0 *array. We first analysed the effect of postnatal age on mRNA regulation by comparing the two SNI groups: young, (PDI) and adult (ADI) rats. We chose seven days post surgery for this analysis as it is known that at this time point, neuropathic behaviour is present in adult but not in young rats [4,16]. The term 'young rats' denotes animals that were 10 days old (P10) at the time of surgery, but whose DRGs were collected for microarray analysis 7 days later, at P17. We used two types of controls to extract specific information obtained from the SNI groups: 1) sham surgery groups in adults (AHI) and young rats (PHI) and 2) contralateral groups in adults (ADC) and young rats (PDC).

Limma analysis was performed to compare the experimental groups in two steps. First, the nerve injury specific gene regulation in adult or young rats was compared to their counterpart sham or contralateral groups to remove those genes that could be up or down-regulated by the surgery alone rather than specifically the nerve injury. The P-value was adjusted for false discovery rate and a threshold of p < 0.05 was set up to subtract those genes with statistically significant differential regulation (Additional file 2). Next, the resulting lists from both the adult and young comparisons were compared to obtain nerve injury specific genes in *adult rats only *(pain behaviour) or *young rats only *(no pain behaviour). The screening provided 206 Affymetrix identifications (Affy_IDs) associated with mRNAs specific to adult SNI rats and only 3 Affy_IDs specific to young (P10) SNI.

**Figure 2:**
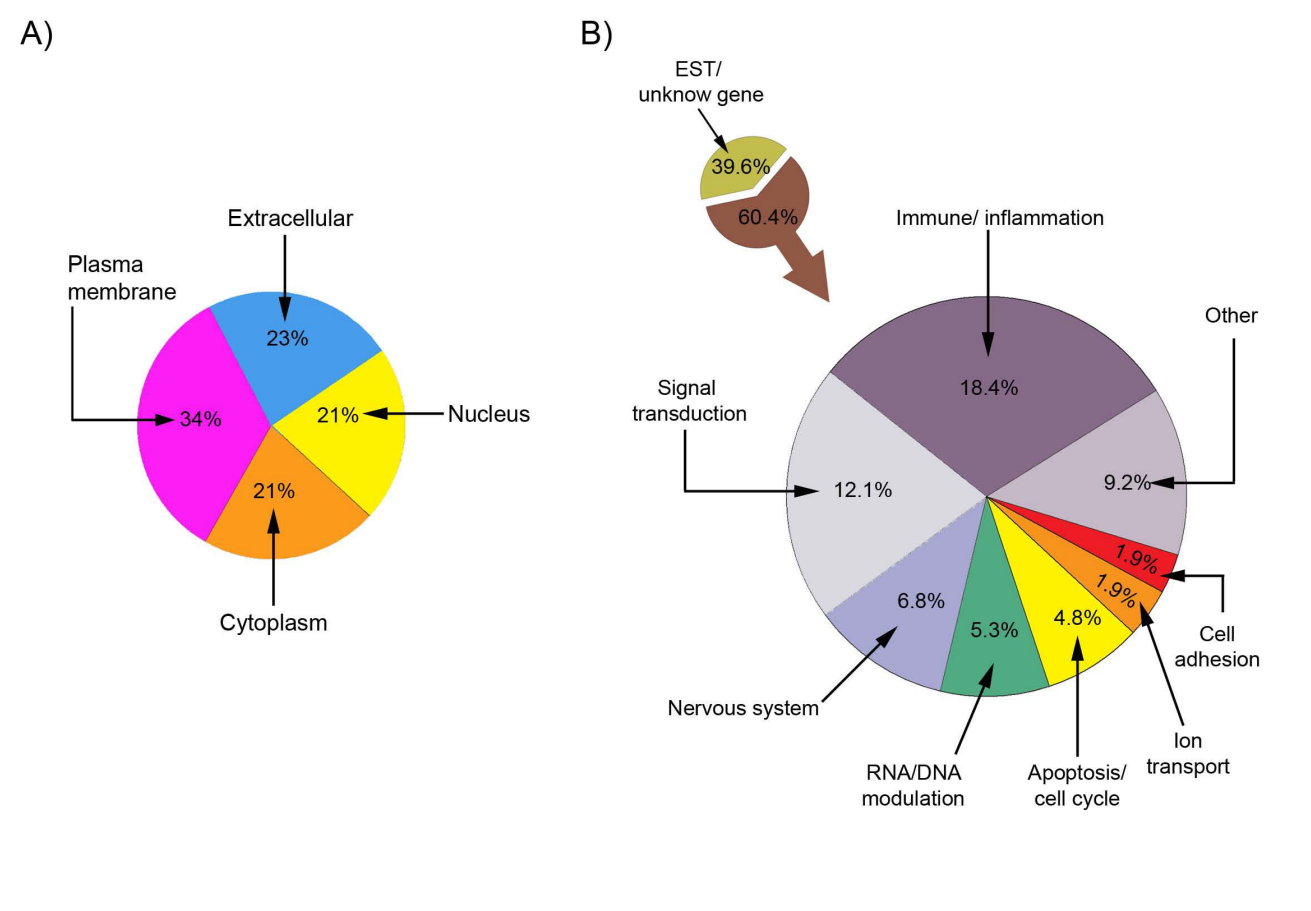
**Cellular location (A) and functional groups (B) of the mRNA Affy_IDs from adult dorsal root ganglia 7 days post spared nerve injury**. Unknown refers to those Affymetrix entries that are either *unknown *genes or *expressed sequences tag *(ETS) that correspond to "transcribed loci" and "similar-to-genes" mRNAs.

The 206 genes were sorted into basic functional groups according to the "Go biological process" [21]. Figure 2A shows that the 206 Affy_IDs were distributed in approximately equal proportions in extracellular (23%), membrane (34%), nuclear (21%) and cytoplasmatic (21%) compartments. We also grouped the genes accordingly to "GO terms" (Figure 2B). Of the 206 genes, 39.6% (82 Affy_IDs) were related to *Express Sequence tags (*EST) (for predicted genes or similarities to known genes) and unknown genes and the rest comprised: immune system/inflammation 18.4% (38 Affy_IDs), signal transduction 12.1% (25 Affy_IDs), nervous system 6.8% (14 Affy_IDs), RNA/DNA modulation 5.3% (10 Affy_IDs), apoptosis/cell cycle 4.8% (10 Affy_IDs), ion transport 1.9% (4 Affy_IDs), cell adhesion 1.9% (4 Affy_IDs) and 9.2% (19 Affy_IDs) related to other processes (additional file 3).

### Neuropathic pain specific gene validation by quantitative Real-time PCR

From the 206 significantly regulated genes, we selected 16 that had some previous published association with pain processes and/or immune system, (Table 2). Using the "Go term" classification, 8 genes belonged to the 'Immune system': (Serine (or cysteine) proteinase inhibitor, clade A, member 3N (SERPINA3N), interleukin 6 (IL-6), colony-stimulating factor 1 (CSF-1), toll-like receptor 4 (TLR4), purinergic receptor P2Y, G-protein coupled, 14 (P2RY14), complement component 3 (C3), macrophage chemoattractant protein 1 (MCP-1/CCL2) and chloride intracellular channel 1 (CLIC-1), one gene belonged to the 'Nervous system': monoamino oxidase A (MaoA); one gene to 'Apoptosis': Caspase 3 (CASP3); two genes to 'Signal transduction': inositol 1,4,5-triphosphate 3-kinase C (ITPKC) and mitogen-activated kinase kinase 6 (MAP2K6); and a further four from other groups: cAMP responsive element modulator (CREM) to the RNA/DNA modulation group and serine (or cysteine) proteinase inhibitor, clade B, member 2 (SERPINB2), matrix metallopeptidase 3 (MMP3) and matrix metalloproteinase 16 (MMP16).

**Table 2:** Summary of putative new gene candidates implicated in the neuropathic pain model SNI at the dorsal root ganglia level.

Affymetrix ID	Gene Name	Gene Symbol	Group	Functionality
1368224_at	Serine (or cysteine) peptidase inhibitor, clade A, member 3N	SERPINA3N	IS	Inflammation [59-61]

1369191_at	Interleukin 6	IL-6	IS	Macrophage in neuropathic pain, inflammation [35]

1379631_at	Colony stimulating factor 1 (macrophage)	CSF-1	IS	Specific factor for macrophages [73-75]

1387982_at	Toll-like receptor 4	TLR4	IS	Inflammation/Immune response/pain [46,47]

1368000_at	Complement component 3	C3	IS	Apoptosis, caspase activation, complement activation, pain processes [50-52]

1367973_at	Macrophage chemoattractant protein-1	MCP-1/CCL2	IS	Chemoattractant, pain processes [41-43]

1370449_at	Purinergic receptor P2Y, G-protein coupled, 14	P2RY14	IS	Immune response [49]

1375633_at	Chloride intracellular channel 1	CLIC-1	IS	Cell cycle progress/Microglia activation [48]

1387690_at	Caspase 3	CASP3	Apop	Apoptosis, inflammation, pain processes [76-78]

1373623_at	Inositol 1,4,5-trisphosphate 3-kinase C	ITPKC	ST	Ca2+ homeostasis [69,79]

1387809_at	Mitogen-activated protein kinase kinase 6	MAP2K6	ST	Modulation of p38 MAPK [62]

1369737_at	cAMP responsive element modulator	CREM	RNA mod	Crem isoform, c-fos regulation, expressed after thermal stimulation [68]

1370678_s_at	Monoamine oxidase A	Maoa	NS	p38/caspase 3 apoptosis pathways [63]

1368657_at	Matrix metallopeptidase 3	MMP3	Other	Remodellation, lymphocytes signalling, inflammation, pain processes [57,80,81]

1368590_at	Matrix metalloproteinase 16	MMP16	Other	Matrix remodelling [54,82]

1368487_at	Serine (or cysteine) proteinase inhibitor, clade B, member 2	SERPINB2	Other	Expressed by DRGs after injury [83]

The table shows the identified gene by Affy_ID, name, gene symbol, Go term classification group and its putative function. IS: immune system; Apop: apoptosis; ST: signal transduction; NS: nervous system; RNA mod: RNA modulation.

**Figure 3:**
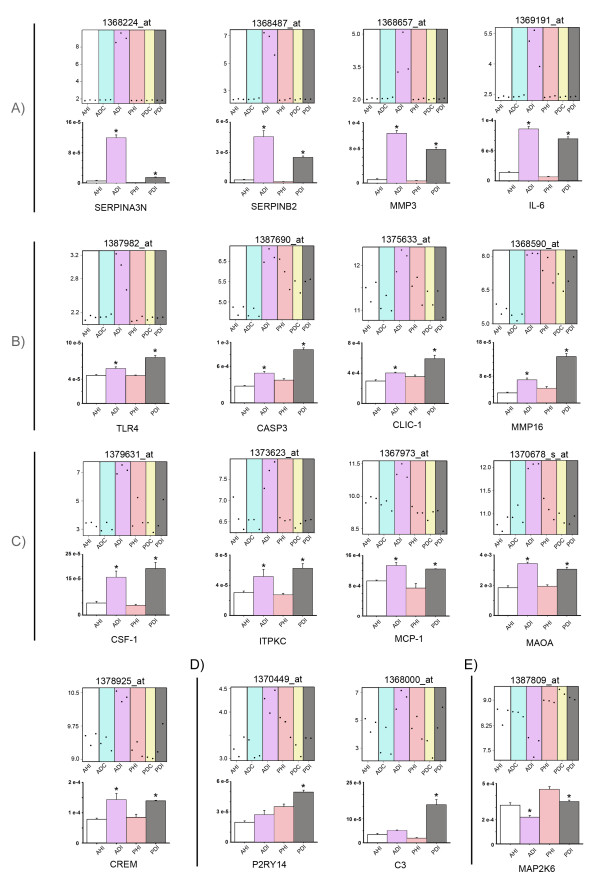
**Microarray and quantitative Real-time PCR (qPCR) validation of genes differentially regulated in the dorsal root ganglia 7 days after SNI surgery**. The figure shows microarray plots of the selected Affy_IDs and counterpart graphs of qPCR validation of the corresponding gene. For clarity, genes have been grouped into A) 'preferentially expressed in adult SNI', B) 'preferentially expressed in young SNI', C) 'genes equally expressed in both adult and young SNI groups', D) 'genes specific to young SNI' and E) 'downregulated gene group'. In the microarray plots, the y-axis indicates the normalised log2 value of expression and each point represents the level of a particular Affy_ID in a single array. In the qPCR validation graphs, the y-axis indicates the mean fluorescence intensity (± standard error). White: Adult sham rats (AHI); Blue: Adult SNI rats, contralateral (ADC); Purple: Adult SNI rats, ipsilateral (ADI); Pink: Young sham rats (PHI); Yellow: young SNI rats, contralateral (PDC); Grey: Young SNI rats, ipsilateral (PDI). For each gene, an ANOVA was performed and p < 0.001. * represent the differences with all experimental groups p < 0.05 in a 'post-hoc' Tukey test or SNK-test, power>80%.

To validate the microarray results of these 16 genes, we used quantitative Real-time PCR (qPCR) and subsequent confirmation of the gene product by DNA sequencing. Figure 3 shows the microarray plot and its counterpart qPCR validation graph for each of the 16 selected Affy_IDs. The qPCR validation shows that the selected genes are differentially regulated in the adult SNI group but many were also regulated in the young SNI group. Overall, 15 genes were upregulated and only 1 gene was downregulated following SNI when compared to sham groups (ANOVA p < 0.001). The 15 upregulated genes were classified into four groups according to the relative expression of the adult versus the young SNI group (Tukey or SNK test p < 0.05) (Figure 3A-D, bar charts). These four groups were A: Genes preferentially expressed in adult SNI: SERPINA3N, SERPINB2, IL-6 and MMP3, B: Genes preferentially expressed in young SNI: CASPASE 3, TLR4, CLIC-1 and MMP16, C: Genes equally expressed in both adult and young SNI: CREM, MCP-1, MAOA, IPTKC and CSF1; and D: Genes specific to the young SNI group: P2RY14 and C3. The fifth group, E, comprises the only downregulated gene, MAP2K6, when compared to the sham groups (ANOVA p < 0.001) (Figure 3E). MAP2K6 was also down regulated in the adult SNI group when compared to the young SNI group (Tukey p < 0.05).

### Immunohistochemical analysis highlights differences in the cellular immune response to nerve injury in young and adult in the DRG

**Figure 4:**
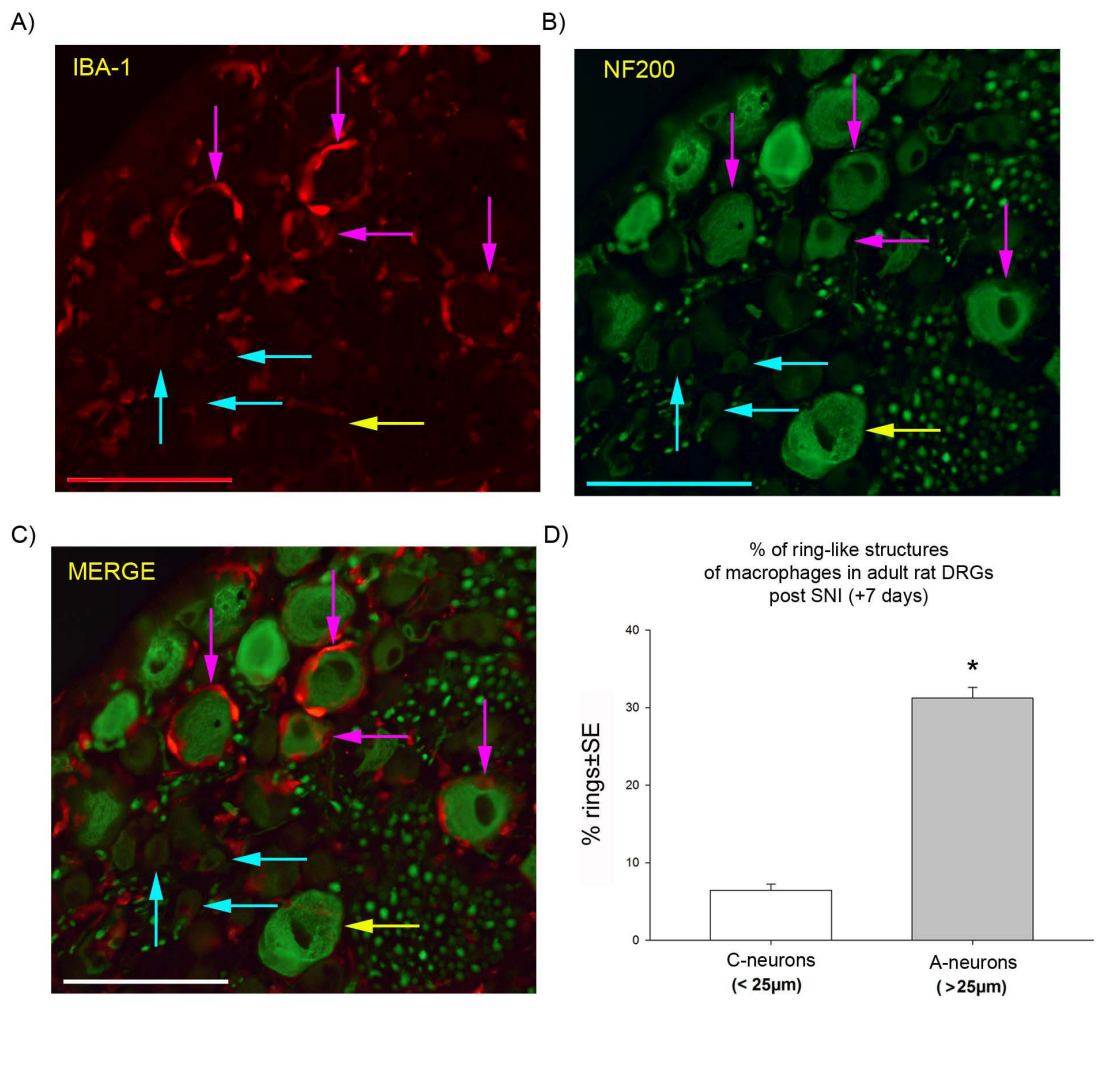
**Reactive macrophages in L4/L5 adult rat dorsal root ganglia 7 days after spared nerve injury**. The immunohistochemistry shows A) macrophages (IBA-1 marker, in red) forming clusters around the large (>25 μm) A-neurons (pink arrows), but not around small C-neurons (blue arrows); B) the same sections stained with NF200 (in green) to show A-neurons, the yellow arrow indicates a large A-neuron without macrophages; C) the merged image of IBA-1 and NF200 staining clearly shows the 'ring-like' structure of macrophages surrounding the A-neurons (pink arrows), while the small C-neurons (< 25 μm) has no macrophage ring (blue arrows); D) a graph showing the percentage of macrophage 'ring-like' formations in DRG neurons 7 days post SNI according to large and small sensory neurons. *: Statistical significance between A- and C-neurons is indicated (t-test, p < 0.001; n = 660 cells, pooled from 3 animals; power> 80%); SE: standard error, scale bar: 100 μm.

**Figure 5:**
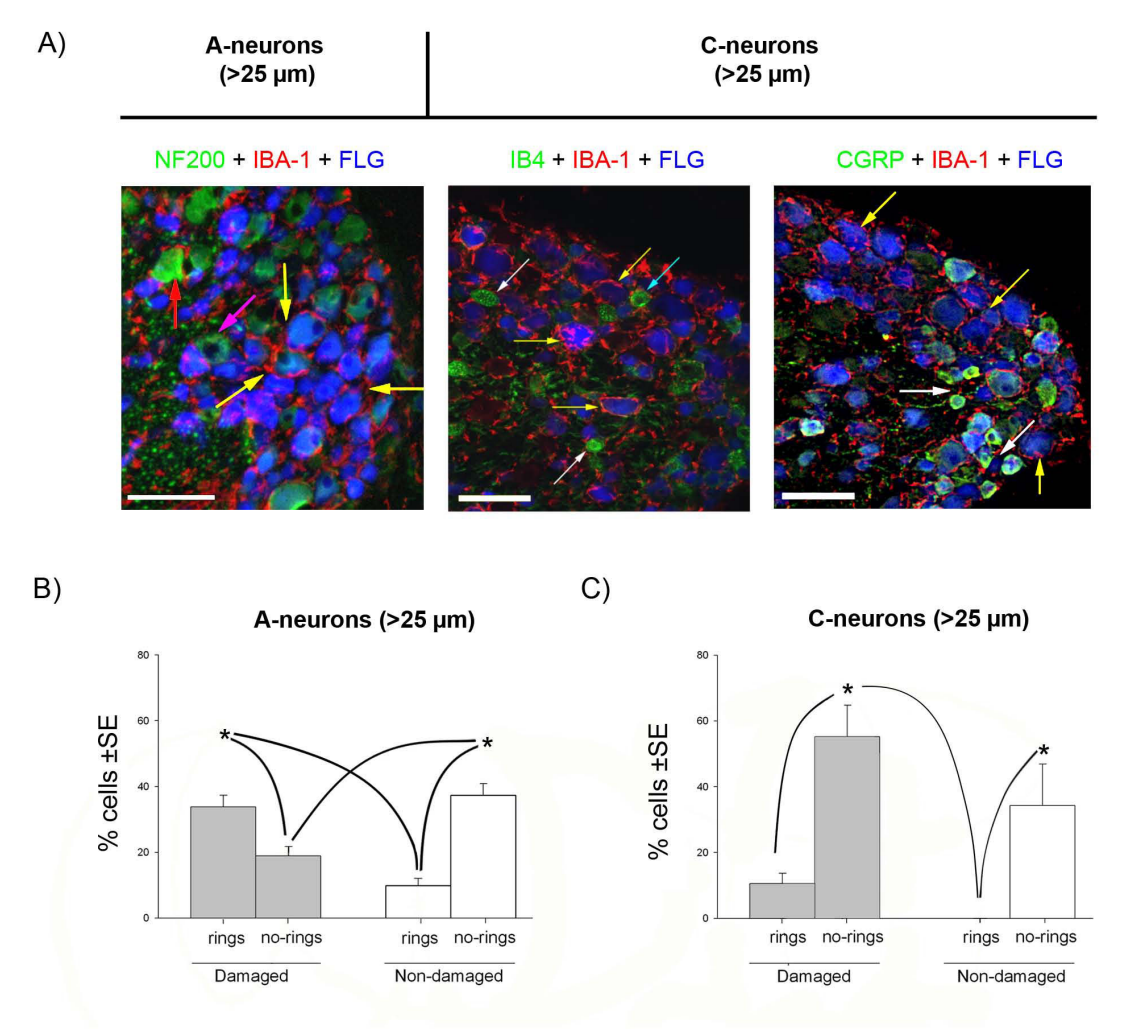
**Analysis of the macrophage invasion of adult dorsal root ganglia 7 days after spared nerve injury**. Fluorogold (FLG, blue) was used as a retrograde tracer injected at the site of nerve injury, to label damaged neurons. A) Macrophages (IBA-1 positive, in red) surround large A-neurons (> 25 μm and NF200 +ve, in green) and are distributed as a 'ring-like' structures. The macrophages have large cell bodies and processes directed toward the damaged neurons (yellow arrows) and undamaged neurons (red arrow). The left panel shows that these 'ring-like' structures surround the many of Fluorogold labelled A-neurons (yellow arrows). Some non-Fluorogold labelled (undamaged) A-neurons are also surrounded by macrophages (red arrow) but others are not (pink arrow). The small diameter (< 25 μm) C-neurons (middle panel: non peptidergic, IB4 +ve, or right panel: peptidergic, CGRP +ve; both in green) do not generally display ring-like displays of macrophages (white arrows) although there are some exceptions (blue arrow). Scale bar: 100 μm. B) Graph showing the relative proportion of damaged (Fluorogold +ve) or undamaged A-neurons (> 25 μm) with macrophage rings (n = 559 A-neurons from 3 animals). C) Graph showing the relative proportion of damaged (Fluorogold +ve) or undamaged in C-neurons (< 25 μm) (n = 284 C-neurons, from 3 animals). Statistical significance is indicated by * (ANOVA p < 0.001, SNK p < 0.05, power>80%). Grey boxes indicate damaged neurons; white boxes indicate non-damaged neurons. Lines indicate the statistical differences between the several experimental groups. SE: standard error.

**Figure 6:**
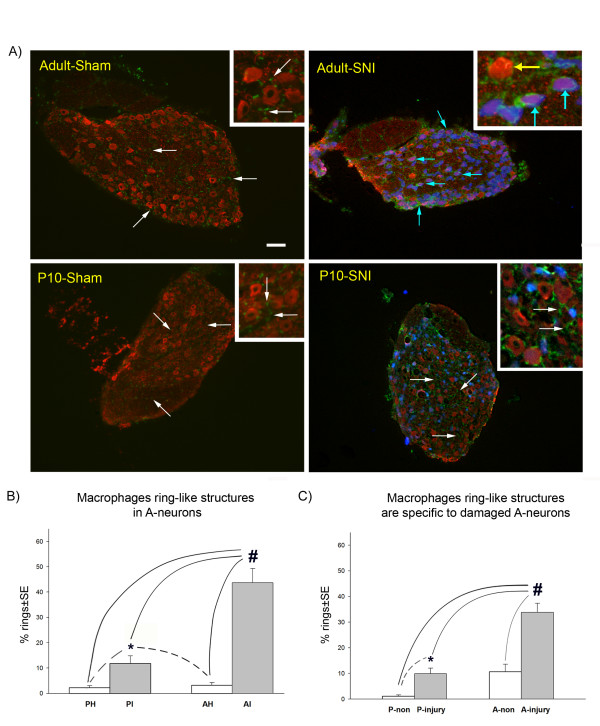
**The effect of postnatal age on the distribution of macrophages in the dorsal root ganglia after spared nerve injury (SNI)**. A) Shows large A-neurons (in red, NF200 +ve cells) and macrophages (in green, IBA-1 +ve) in adult or young rats in sham and injured groups (7 days after nerve injury). Both young and adult sham groups have inactivated macrophages (white arrows). In contrast, in adult animals post injury, the macrophages have large cell bodies (blue arrows) which form a 'ring-like' structure that surround the large, injured A-neurons. The majority of non-injured neurons do not have these rings (yellow arrow). In young SNI animals (P10, 10 days old at the time of nerve injury) the macrophage distribution is similar to the sham groups (white arrows) with a small increase in 'ring-like' structures which is only significant when compared to the sham groups. B) Counts of 'ring-like' structures in young and adult DRG 7 days post SNI surgery. PI: ipsilateral SNI surgery at 10 days old; PH: sham at 10 days old, AI: ipsilateral to SNI surgery in adult; AH: sham in adult. *: indicates statistically significance when compared to the sham groups; #: indicates statistical significance when compared to all experimental groups (ANOVA p < 0.001; SNK p < 0.05; n = 2219 A-neurons from 3 animals; power>80%). C) Counts of 'ring-like' structures in damaged or non-damaged neurons in adult and young SNI operated animals. A preferential attraction of macrophages towards damaged large A-neurons is evident in the adult SNI group. P-non: non damaged A-neurons in young rats; P-injury: damaged A-neurons in young rats; A-non: non damaged A-neurons in adult rats; A-injury: damaged A-neurons in adult rats. Fluorogold (in blue) was used as a retrograde tracer injected at the site of the injury, to label damaged neurons. #: indicates statistical significance in all experimental groups (ANOVA p < 0.001; SNK p < 0.05; n = 559 A-neurons from 3 animals; power>80%) *: indicates statistical significance (t-test p < 0.005) when an independent t-test was performed on the young groups only (dashed lines). SE: standard error. Scale bar: 100 μm.

Since many of the differentially regulated genes between young and adult SNI were related to the immune system, we compared the cellular neuroimmune reaction in the DRG following nerve injury at the two ages. We hypothesised that if the differing patterns of gene regulation had functional implications we might see differences in injury induced reactions of the major cellular component of the immune system in peripheral ganglia, the macrophages. To determine macrophage reactions in the DRG after nerve injury we immunostained them with the macrophage marker IBA-1 in combination with selective markers of subpopulations of DRG sensory neurons, NF200 (for A-neurons), CGRP and IB4 (for C-neurons), 7 days following SNI surgery in young and adult rats (Figures 4, 5 and 6).

Figure 4 shows the striking macrophage response in adult rats 7 days post SNI surgery. Macrophages can be seen (in red) clustering around neuronal cell bodies in characteristic 'ring-like' structures with processes extending from their enlarged cell bodies towards neurons (Figure 4A pink arrows). Figures 4B and 4C show that the macrophages are selectively clustered around the large A-neurons which are stained with the marker NF200 (in green) (Figure 4B, pink arrows) and not around the C-neurons (Figure 4, blue arrows). We have confirmed these results by double labelling IBA-1 +ve macrophages in these 'ring-like structures with CD68/ED-1, another specific marker of macrophages [22] (additional file 4). NG2, on the other hand, a specific marker of satellite cells [23], did not overlap with IBA-1(additional file 5). As C-neuron immunomarkers are down-regulated after nerve injury [24-26], these could not be used to quantify macrophage clustering, but instead neuronal diameter was used, The analysis showed that 'rings' of macrophages are preferentially (p < 0.01, t-test) located around large (> 25 μm) NF200 +ve A-neurons and that smaller (< 25 μm) C-neurons are relatively free of macrophages. Figure 4D shows that 37.6% of neurons from adult DRG have macrophage rings around them 7 days post SNI (n = 660 cells pooled from 3 animals). Of these, 31.2% surround large A-neurons while 6.4% surround small C-neurons.

To investigate whether macrophage reactivity was restricted to neurons whose axons were directly damaged in the SNI surgery or whether neurons with 'spared' intact axons also display 'rings' of macrophages, we distinguished damaged from non-damaged neurons in adult L4 and L5 DRG using a retrograde tracer. Fluorogold was injected into the injured nerves (tibial and common peroneal) at the time of surgery and subsequently counterstained with the neuronal markers NF200, IB4 or CGRP (Figure 5A). We then counted 'ring-like' structures around damaged and non-damaged neurons and correlated the data with cell body diameter. Of the total population of large A-neurons in adult L4 and L5 DRGs, 7 days post SNI, 52.8% were damaged (Fluorogold +ve) and 47.2% were non-damaged (Fluorogold -ve) (n = 559 A-neurons form n = 3 animals). The data shows that the proportion of 'ring-like' structures in damaged A-neurons is significantly greater than in undamaged A-neurons: 33.8% of damaged A-neurons have 'ring-like' structures compared to 9.9% of the non-damaged A-neurons (Figure 5B) (ANOVA p < 0.001). In contrast, only 10% of the damaged C-neurons have 'ring-like' structures, while the non-damaged C-neurons have none (Figure 5C) (n = 284 C-neurons pooled form from 3 animals).

In young DRGs, however, the macrophages rarely formed 'rings' around neurons but remained scattered throughout the DRG (Figure 6A, white arrows). Thus, the distribution of macrophages in DRGs from young rats subjected to SNI surgery resemble that of adult and young sham groups with small cell bodies and few processes (Figure 6A white arrows). Figure 6B shows that only 11.7% of large A-neurons have macrophage 'ring-like' formations in the young SNI group, which although significantly greater than sham (1-2%), is significantly less than the adult SNI group, where 43.7% of the large A-neurons display 'rings' of reactive macrophages (ANOVA, p < 0.001; SNK p < 0.05, n = 2219 A-neurons from 3 animals, Figure 6B). Figure 6C compares the damaged and non-damaged neuronal groups in adult and young rats with SNI surgery. While in adult DRGs the macrophage 'ring-like' structures occur in 33.8% of damaged large A-neurons and 9.9% of non-damaged A-neurons, in young DRGs, significantly fewer A-neurons have macrophage 'ring-like' structures (10.6% of damaged, 1.1% of non-damaged) (Figure 6C). (n = 559 A-neurons from 3 animals)

## Discussion

In this study, we have used differential Affymetrix screening to examine age-related differences in transcriptional changes in the dorsal root ganglion (DRG) of young and adult rats after partial nerve damage "spared nerve injury" model [16]). The study was triggered by the absence of neuropathic behaviour following nerve injury in young rats and humans in contrast to the hypersensitivity and spontaneous pain that develops after adult nerve injury [1]. Our results show a very high level of transcriptional activity in DRGs following nerve injury in adulthood compared to nerve injury in early life. Validation experiments provided a list of genes, normally implicated in the regulation of the immune system (Table 2), which have a specific and different pattern of expression in the DRGs of adults and young rats. We have also used immunohistochemical analysis to reveal a major differential reactivity in DRGs macrophages, the cellular component of immune responses. Reactive macrophages cluster in a characteristic display around the large A-neurons in adult neuropathic DRGs while little difference is found in the pattern of macrophages in young rats. Together, our results suggest a strong correlation between the regulation of the peripheral nervous system immune system and the progression towards neuropathic pain.

The microarray analysis results provided 206 candidate genes that were statistically significantly regulated in adult rats only. Intriguingly, only 3 genes were differentially regulated in young animals after nerve injury and no overlap was observed between the two age groups. The absence of neuropathic pain behaviour at 7 days post nerve injury in young rats [4,5] correlates with this low level of differentially modulated genes in the young rat DRGs at the same time point.

On validating those Affy_IDs related to adult neuropathic pain, we found that most genes were closely related to the immune system/inflammation, followed by genes related to signal transduction, the nervous system and RNA/DNA modulation. It is important to note that the highest proportion (39%) of Affy_IDs (82 entries) belong to a group of unknown "transcribed loci" or "EST" (*Expressed Sequence Tags*). Other microarray analyses of the DRG transcriptome have highlighted the differential regulation between different experimental neuropathic models using total or partial axotomy [27-30]. Here we present a whole new set of "molecular tools" as new targets to dissect out the peripheral mechanisms in the 'spared nerve injury' model of neuropathic pain.

Several (16 Affy_IDs) of the top gene candidates from the microarray analysis were validated using quantitative real-time PCR (qPCR). Interestingly, the majority of these genes (15 Affy_IDs) were also differentially regulated in young animals after nerve injury. The difference between the initial microarray results and the qPCR data arises because microarray studies show widespread variations, even under the same experimental conditions and it is essential that the results are confirmed by other independent methods [31]. Thus, qPCR highlights small differences not recognised by microarray technology. In addition, our gene lists were subjected to a stringent p-value correction for "false discovery rate" in the Limma test, designed to minimize false positive candidates [32]. Overall these results suggest that the young DRG is not simply regulating the same genes less efficiently than in the adult but rather there is a different pattern of regulated genes in early life.

Our data reveals an interesting set of genes which are implicated in the modulation of the immune system. Thus, we have found a set of chemokines which are upregulated in the dorsal root ganglia after SNI nerve injury such as interleukin-6 (IL-6), macrophage chemoattractant protein-1 (MCP-1/CCL-2), colony-stimulating factor 1 (CSF-1) and complement component 3 (C3). Proinflammatory cytokines have a recognized role in neuropathic pain [33]. Particularly well-studied is IL-6, which is upregulated in the DRG following chronic constriction injury and when blocked by minocycline reverses hyperalgesia and allodynia [34]. IL-6 is upregulated in several microarray analyses of other models of axotomy [27,30] and is associated with macrophage invasion in peripheral nerve [35]. Spinal administration of IL-6 has anti-nociceptive effects in the spinal nerve ligation neuropathic pain model measured by a dose-related inhibition of electrically evoked C-fibre activity [36]. MCP-1 is a chemokine that plays a role in the recruitment of monocytes to sites of injury and infection [37] and a role in pain processes [38]. Exogenous administration of MCP-1 is known to depolarize and evoke action potential activity in DRG [39] and to excite cultured neonatal DRG neurons and to promote the secretion of the neuropeptides, substance P and CGRP [40,41]. Moreover, MCP-1 receptor CCR2 knockout mice fail to develop mechanical allodynia after nerve injury [38]. As for immune system activation and recruitment, MCP-1 is associated with both resident spinal microglia and infiltrating macrophages [42] and CCR2 null mice do not develop microglial activation [43]. Nerve injury promotes the expression of MCP-1 in DRG cells [44] and it is possible that it is released centrally in neuropathic pain states. CSF-1 also has a role in microglia activation [45]. The upregulation of these three genes observed here in the DRG suggests a similar role as described in the spinal cord. Since macrophage activation in the DRG after nerve injury is greater in adult than in young SNI rats, chemokine modulation could be a key factor underlying the different neuropathic pain behaviour at the two ages.

Other validated genes support the role of macrophage activation in adult DRGs, such as toll-like receptor 4 (TLR4) [46,47] and the chloride intracellular channel 1 (CLIC-1) [48]. Interestingly, in young injured DRGs we have found other markers related to macrophage activity such as the chemotactic purinergic receptor P2Y, G-protein coupled 14 [49] and complement component 3 [50-52] suggesting a differential macrophage activity, rather than simple absence of activity through the postnatal development.

A further group of regulated genes in DRGs after nerve injury have a general role in regulating the extracellular matrix environment. Thus, matrix metallopeptidases 3 and 16, (MMP3 and MMP16) are proteoglycanases capable of degrading the major components of the extracellular matrix [53,54] and are related to macrophage invasion [55]. Recent studies have shown that other metalloproteinases, MMP2 and MMP9 contribute to the allodynia that follows neuropathy [56]. MMP3 has recently been implicated in the inflammatory response [57] and the immune reaction in some neuropathies [58]. Possibly counteracting the metalloproteinases, we found two genes: serine (or cysteine) inhibitor, clade A, member 3N (Serpin A3) and serine (or cysteine) inhibitor, clade B, member 2 (Serpin B2) that are implicated in protease inhibition and inflammation [59-61] which are specific for the adult SNI group. This data suggest that the balance of these particular MMPs and Serpins may be essential for macrophage invasion in the DRGs after nerve injury.

The final group of validated genes was related to intracellular signalling pathways such as Mitogen-activated protein kinase kinase 6 (MAP2K6) and Monoamino oxidase A (MAOA) which are related to the modulation of the p38 pathway [62,63]. The p38 pathway has a role in microglia activation in the spinal cord [33,64] and so this data suggests two new putative candidate targets for macrophage modulation as part of the p38 cascade of activation. Caspase 3, known to be involved in apoptosis [65] and cell loss [66] and TLR4 modulation [67] are upregulated in both adult and young SNI group, which is consistent with the stress produced by the nerve injury. cAMP responsive element modulator (CREM) isoform (ICER) has a demonstrated role in c-fos regulation after thermal stimulation and its overexpression represses the c-fos and c-jun promoters and attenuates early gene induction following noxious stimulation [68]. Inositol 1,4,5-trisphosphate 3-kinase C (ITPKC) has been implicated in Ca2+ homeostasis and T lymphocytes activity [69], its upregulation after the nerve injury suggests a possible role in the lymphocyte signalling.

Our microarray analysis highlights the importance of the immune system in the SNI neuropathic model and corroborates the relationship between the activation of both microglia and macrophages and neuropathic pain behaviour [9,14]. Nevertheless, it is clear that neuropathic pain behaviour is not simply a result of macrophage activation: some genes related to macrophage/microglia reactivity are upregulated in young rats after nerve injury despite the fact that they have no neuropathic pain response [4]. Immunohistochemical analysis suggests that equally important is the pattern of macrophage response: in the adult rat post injury, activated macrophages take on a characteristic [70,14] distribution throughout the DRGs, selectively surrounding the large A-neurons in a 'ring-like' structures. In contrast, macrophages in young rats post injury do not form these 'ring-like' structures and are not selectively recruited to the injured A-neurons. This observation suggests that the failure of macrophages in young animals to be attracted toward damaged A-neurons could be a key factor responsible for the lack of neuropathic behaviour. This could be due to the absence of released macrophage activation/chemoattractant factors. Our results are consistent with those of, Murphy et al, 1996 who described the presence of IL-6 in large and medium size neurons in axotomized adult DRGs [71]. Hence, the immature neuroimmune response to nerve injury and the consequent lack of neuropathic pain in young mammal may not be due to a deficient immunological capacity per se but to antigenic inexperience [72] which leads to macrophages failing to specifically target neurons in the DRGs.

## Conclusions

Differential microarray analysis has revealed clear differences in the response in the dorsal root ganglia to peripheral nerve injury in adult and young rats which are likely to underlie the known age related differences in the onset of neuropathic pain. Genes involved in the immune system, many of which play a role in macrophage activity, are the most differentially regulated group following adult nerve injury relative to the younger animals. Consistent with this, macrophages are activated in the adult DRGs, in a characteristic pattern around large A sensory neurons following nerve injury but this pattern of activation does not occur in young rats. This study highlights the important contribution of the maturity of the immune system and neuron/macrophage interactions to the postnatal development of neuropathic pain.

## Abbreviations

SNI: Spared Nerve Injury; ADI: Adult SNI group, ipsilateral side; ADC: Adult SNI group, contralateral side; AHI: Adult sham group, ipsilateral side; PDI: Young SNI group, ipsilateral side; PDC: Young SNI group, contralateral side; PHI: Young sham group, ipsilateral side; DRGs: Dorsal Root Ganglia.

## Competing interests

The authors declare that they have no competing interests.

## Authors' contributions

DVA designed the study, carried out all the experiments, performed the statistical analysis and wrote the manuscript. SMG assisted with the Affymetrix data analysis. MF is the director of the research and wrote the manuscript. All authors have read and approved the final manuscript.

## Supplementary Material

Additional file 1 **Colocalisation of ATF-3 and Fluorogold in damaged neurons of adult DRGs**. To quantify damaged neurons we used the retrograde tracer Fluorogold (see 'Methods' sections). The figure represents the validation of our technique. ATF-3 (in red), is a marker of neuronal damage [18] and overlaps with Fluorogold staining (in blue) in 92.4% (± 1SE) of the neurons. Yellow asterisks indicate colocalisation of ATF-3 and Fluorogold; white asterisks indicate undamaged neurons with no immunoreactivity for both markers. Confocal images; optical sections: 0.8 μm, Scale bar: 30 μm. Click here for file

Additional file 2 **Microarray Comparison Table**. This spread sheet file that contains the microarray data used obtained from the Limma test analysis. It contains the Affymetrix identification number (Affy_ID), the fold difference in log2 format of the comparison and the adjusted p-value. There are several columns that contain: the ipsilateral side of the adult SNI (ASI) versus (vs) either the contralateral side (ASC) or the sham group (ASH); and the ipsilateral side of the young SNI (PSI) versus (vs.) either the contralateral side (PSC) or the sham group (PSH). Those entries with p < 0.05 are on a white background and those with p > 0.05 are on a grey background. The original '.cel' files used for the analysis can be accessed at 'Gene Expression Omnibus (GEO)' database with accession number: GSE15041 http://www.ncbi.nlm.nih.gov/projects/geo/query/acc.cgi?acc=GSE15041. Click here for file

Additional file 3 **Summary Gene Classification**. This spread sheet file has the gene classification for those genes specifically modified in the adult SNI group. The columns are: the gene group, the Affy_IDs, gene symbol, gene name and the "Gene ontology" (GO) term(s) that classify the gene. Click here for file

Additional file 4 **Macrophage ring formation around adult DRGs neurons 7 days after nerve injury**. A-D) show reactive macrophages in adult nerve damaged DRGs which upregulate and coexpress the specific macrophage markers CD68/ED-1 (in green, A) and IBA-1 (in red, B). These macrophages (white arrow heads) infiltrate and form a 'ring-like' structure around the damaged neurons which are shown in (C) in blue, labelled with Fluorogold. (D) Note the colocalisation of the CD68 and IBA-1(in yellow) in the macrophage ring formations. (E & F) In adult sham DRGs, the macrophages (yellow arrow heads) are not stained with CD68 (in green) although resting macrophages can be identified with IBA-1 (in red). Confocal images; optical sections: 0.8 μm, Scale bar: 30 μm. Click here for file

Additional file 5 **Satellite cells and macrophage staining in adult DRGs**. The satellite cells (yellow arrow heads) are labelled with the specific marker NG2 (in green, A, D, E and G) and the macrophages (white arrow heads) with IBA-1 (in red B, D, F and G). We found no colocalisation of these two distinctive markers of satellite cells (NG2) and macrophages (IBA-1). A - D show cells from adult DRGs after nerve injury. C) shows neurons labelled with the retrograde tracer Fluorogold (in blue) which are surrounded by infiltrated macrophages (B and D). E - G show cells from adult sham DRGs where the macrophages have small cell bodies and are scattered through the section. Confocal images; optical sections: 0.8 μm, Scale bar: 30 μm. Click here for file
