# Supplementary figures and images for: Differential regulation of immune responses and macrophage/neuron interactions in the dorsal root ganglion in young and adult rats following nerve injury
Source: Mol Pain. 2009 Dec 10;5:70. doi: 10.1186/1744-8069-5-70 (PMC2799401; doi:10.1186/1744-8069-5-70)

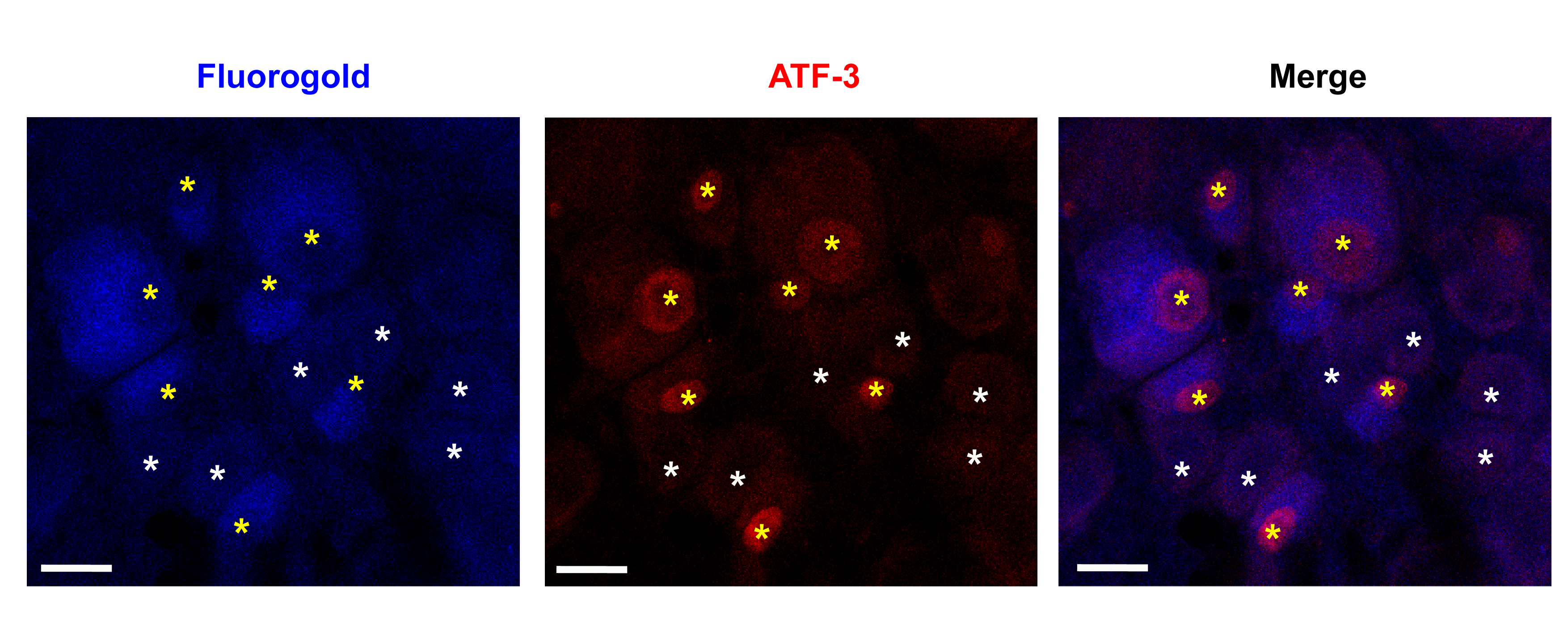

Supplement: Additional file 1 — Colocalisation of ATF-3 and Fluorogold in damaged neurons of adult DRGs. To quantify damaged neurons we used the retrograde tracer Fluorogold (see 'Methods' sections). The figure represents the validation of our technique. ATF-3 (in red), is a marker of neuronal damage [18] and overlaps with Fluorogold staining (in blue) in 92.4% (± 1SE) of the neurons. Yellow asterisks indicate colocalisation of ATF-3 and Fluorogold; white asterisks indicate undamaged neurons with no immunoreactivity for both markers. Confocal images; optical sections: 0.8 μm, Scale bar: 30 μm. [file 1744-8069-5-70-S1.TIFF]

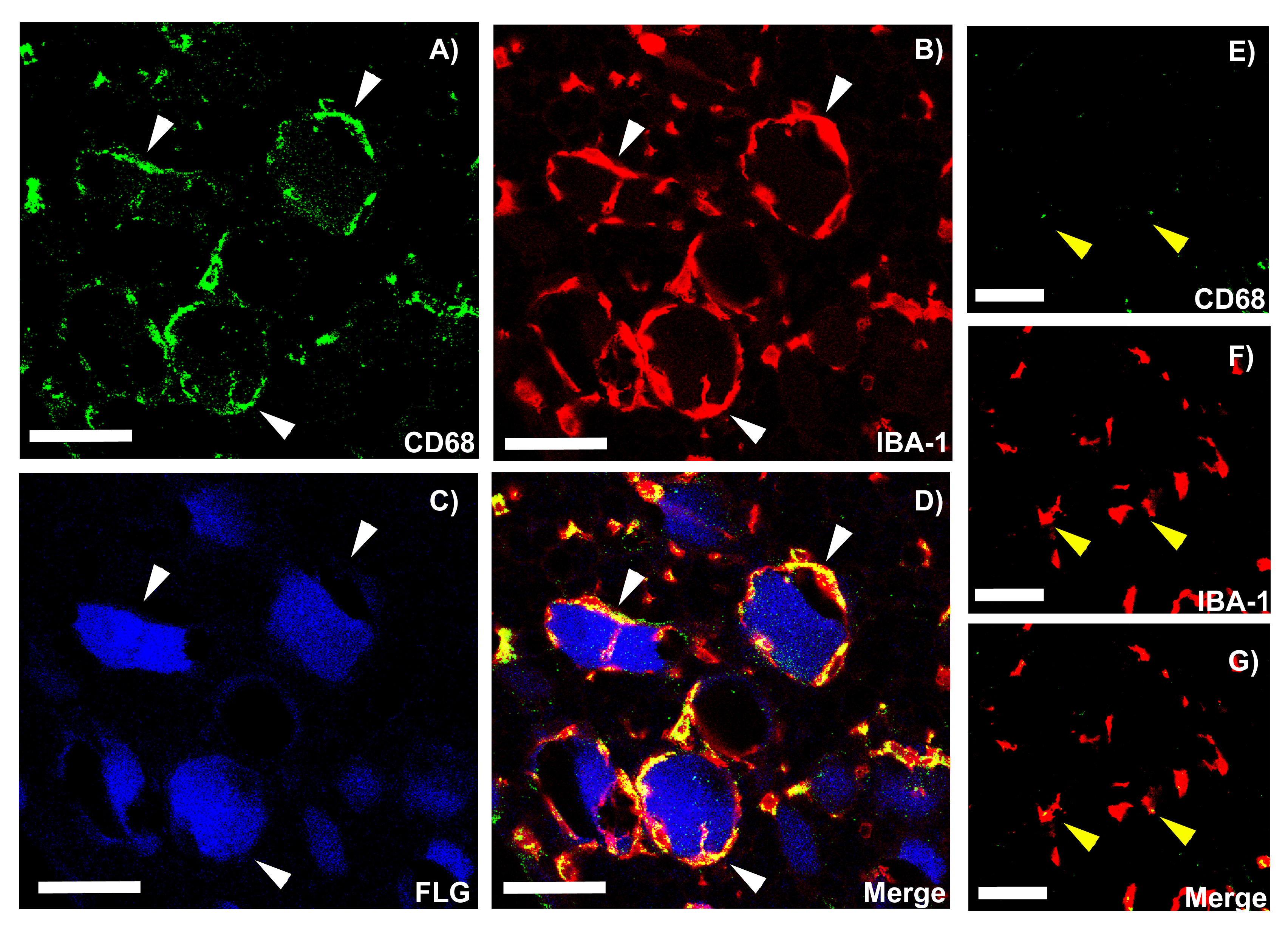

Supplement: Additional file 4 — Macrophage ring formation around adult DRGs neurons 7 days after nerve injury. A-D) show reactive macrophages in adult nerve damaged DRGs which upregulate and coexpress the specific macrophage markers CD68/ED-1 (in green, A) and IBA-1 (in red, B). These macrophages (white arrow heads) infiltrate and form a 'ring-like' structure around the damaged neurons which are shown in (C) in blue, labelled with Fluorogold. (D) Note the colocalisation of the CD68 and IBA-1(in yellow) in the macrophage ring formations. (E & F) In adult sham DRGs, the macrophages (yellow arrow heads) are not stained with CD68 (in green) although resting macrophages can be identified with IBA-1 (in red). Confocal images; optical sections: 0.8 μm, Scale bar: 30 μm. [file 1744-8069-5-70-S4.TIFF]

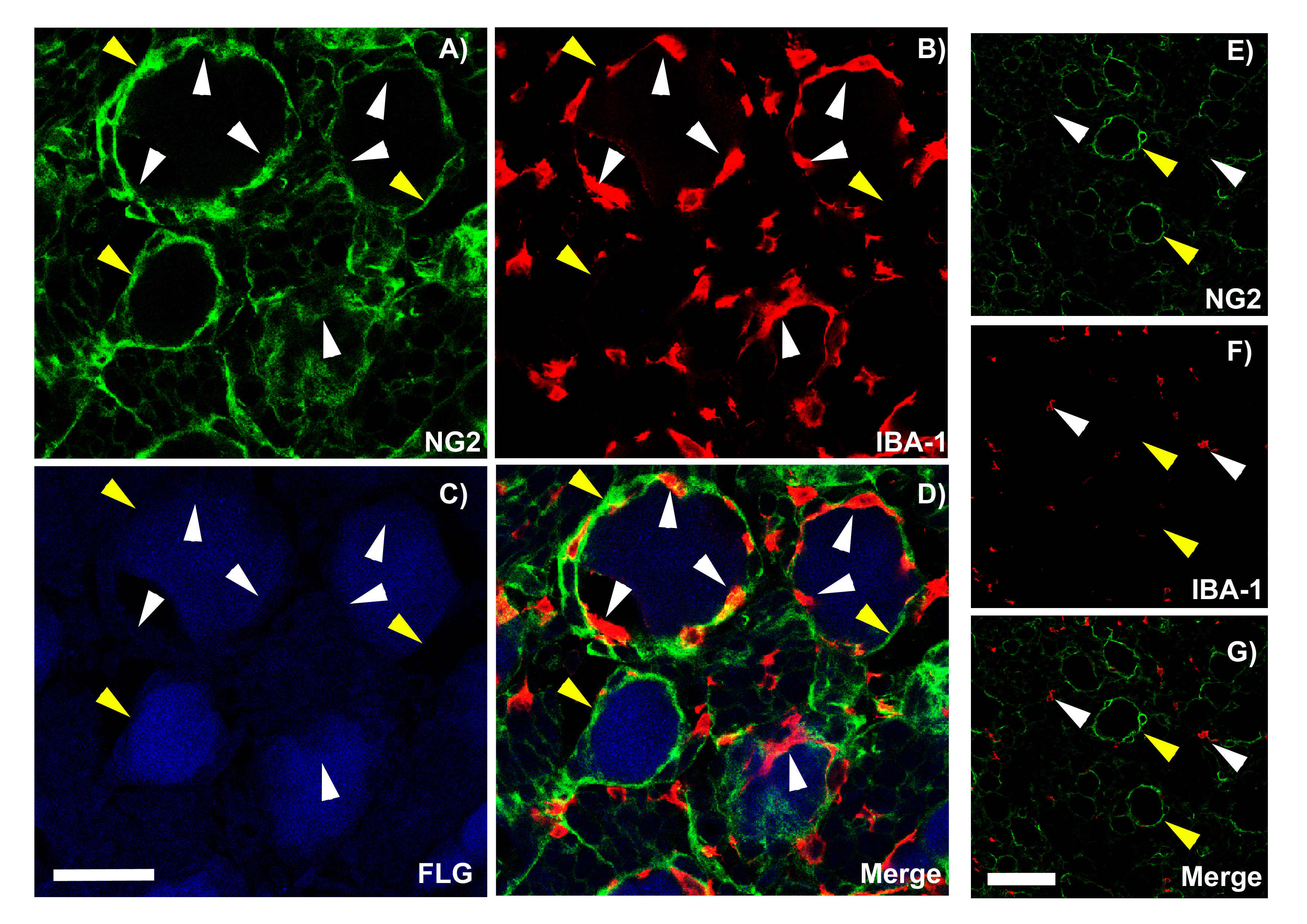

Supplement: Additional file 5 — Satellite cells and macrophage staining in adult DRGs. The satellite cells (yellow arrow heads) are labelled with the specific marker NG2 (in green, A, D, E and G) and the macrophages (white arrow heads) with IBA-1 (in red B, D, F and G). We found no colocalisation of these two distinctive markers of satellite cells (NG2) and macrophages (IBA-1). A - D show cells from adult DRGs after nerve injury. C) shows neurons labelled with the retrograde tracer Fluorogold (in blue) which are surrounded by infiltrated macrophages (B and D). E - G show cells from adult sham DRGs where the macrophages have small cell bodies and are scattered through the section. Confocal images; optical sections: 0.8 μm, Scale bar: 30 μm. [file 1744-8069-5-70-S5.TIFF]
